# Advances Achieved by Ionic-Liquid-Based Materials as Alternative Supports and Purification Platforms for Proteins and Enzymes

**DOI:** 10.3390/nano11102542

**Published:** 2021-09-28

**Authors:** Rui M. F. Bento, Catarina A. S. Almeida, Márcia C. Neves, Ana P. M. Tavares, Mara G. Freire

**Affiliations:** CICECO-Aveiro Institute of Materials, Department of Chemistry, University of Aveiro, 3810-193 Aveiro, Portugal; ruib@ua.pt (R.M.F.B.); ac.almeida@ua.pt (C.A.S.A.); mcneves@ua.pt (M.C.N.); aptavares@ua.pt (A.P.M.T.)

**Keywords:** ionic liquids, supported ionic liquids, supports, purification platforms, chromatography, proteins, enzymes, biocatalysis

## Abstract

Ionic liquids (ILs) have been applied in several fields in which enzymes and proteins play a noteworthy role, for instance in biorefinery, biotechnology, and pharmaceutical sciences, among others. Despite their use as solvents and co-solvents, their combination with materials for protein- and enzyme-based applications has raised significant attention in the past few years. Among them, significant advances were brought by supported ionic liquids (SILs), in which ILs are introduced to modify the surface and properties of materials, e.g., as ligands when covalently bond or when physiosorbed. SILs have been mainly investigated as alternative supports for enzymes in biocatalysis and as new supports in preparative liquid chromatography for the purification of high-value proteins and enzymes. In this manuscript, we provide an overview on the most relevant advances by using SILs as supports for enzymes and as purification platforms for a variety of proteins and enzymes. The interaction mechanisms occurring between proteins and SILs/ILs are highlighted, allowing the design of efficient processes involving SILs. The work developed is discussed in light of the respective development phase and innovation level of the applied technologies. Advantages and disadvantages are identified, as well as the missing links to pave their use in relevant applications.

## 1. Introduction

Ionic liquids (ILs) have attracted enormous interest over time, with their boost verified in the 21st century. ILs have been considered as a novel generation of materials and solvents, depending on the objective of their application [1]. These compounds are ionic species formed by an organic cation and a small inorganic or organic anion [2,3]. Ionic liquids are organic salts with a lower melting temperatures than inorganic salts, which can be liquid at amenable temperatures and as such be applied as solvents, being a main result of their asymmetric and large chemical structures that hinder their crystallization [1]. However, in contrast to most molecular solvents, and if properly designed, ILs display low flammability, negligible vapor pressure, high chemical and thermal stability, high conductivity, as well as a wide electrochemical window [1,4].

Due to their myriad of chemical structures, ILs present a vast spectrum of physicochemical properties, which has led them to be extensively used in diverse areas of science [5]. They have been applied as substitutes to traditional solvents in chemical synthesis [5], (bio)catalysis [6,7,8], and separation [9,10] and extraction processes [11], which is reflected by significant and an ever-increasing literature in these fields [3,12]. On the other hand, supported ionic liquids (SILs), in which ILs are immobilized on a solid support, are among the most important derivatives of ILs [13]. They merge the distinctive properties of ILs with the advantages exhibited by materials, resulting in functionalized or modified materials with designed performance to target applications [14,15]. 

Proteins are macromolecular polypeptides with application in biochemical, biotechnology, chemical, food and pharmaceutical industries [16]. However, their biological function and activity, which is associated with the three-dimensional structure, can be disrupted through modifications in the medium temperature, pH, or composition [17]. Thus, the preservation of the structure of proteins in their applications is the main challenge, which may be achieved by favoring particular interactions, for instance hydrogen bonding, hydrophobic, and electrostatic forces with a solvent or material [18]. Several approaches such as immobilization, chemical modification, or genetic modification have been established to carry out the stabilization of proteins out of their native environments and improve their biological functions. ILs have been investigated in this field as well, with a large fraction of the respective publications dealing with the interactions of ILs with proteins, while seeking the fundamental knowledge and factors that improve their stability in the different IL environments. Other scientific fields involving proteins and ILs deal with proteins’ solubility and separation [19]. 

**Figure 1:**
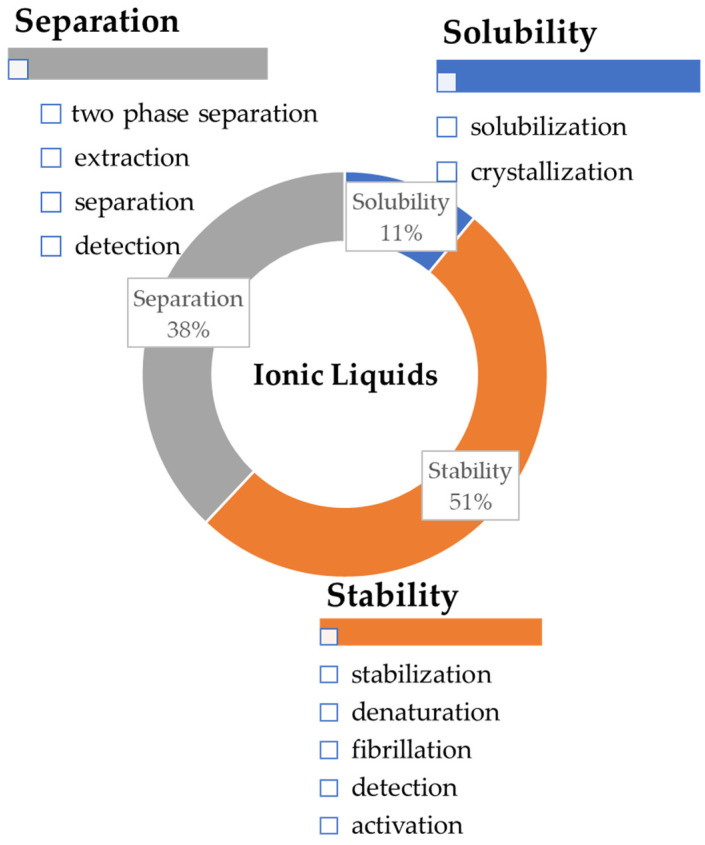
Publications between 2009 and 2020 about the use of ILs in different protein and enzyme applications using the following keywords: “ionic liquids”, “enzyme”, “proteins”, and “applications” (Source of Information: ISI Web of Knowledge, https://www.webofscience.com/wos/woscc/basic-search, accessed on 30 December 2020).

Figure 1 summarizes the relative amount of works found in the literature, between 2009 and 2020, involving ILs and proteins/enzymes. Despite the shown division, it should be kept in mind that crystallization can be included in the separation category, whereas stability is mainly applied in works involving enzymes and biocatalysis, which are discussed below.

Enzymes are biocatalysts, i.e., proteins that have biocatalytic properties [20]. Most enzymes are normally not stable and exhibit a low solubility in solvents other than aqueous buffered conditions [21]. As a consequence of the high industrial and technological applicability of these biological molecules, the search for the improvement of their enzymatic activity is an ongoing practice [19,22]. Currently, a large fraction of enzymatic catalysis is performed at high temperature, where the yield of the obtained product is elevated [21,23]. Thereby, the enzyme’s stability must be ensured at the required temperatures and using appropriate solvents, in which ILs have been shown to lead to significant improvements [17,24].

Up to date, noteworthy developments have been achieved by the application of ILs in biocatalytic processes [17,25]. Their ability to provide favorable effects on the catalytic performance of enzymes compared to what occurs in traditional solvents or in the native aqueous or non-aqueous reaction medium of the enzyme has been disclosed [26]. The referred effects comprise an enhanced rate of reaction and conversion, as well as improvements in enantioselectivity and regioselectivity [3,27]. In addition, ILs allow different intermolecular interactions, facilitating enzymatic transformations of poorly soluble substrates in organic solvents [3]. Accordingly, numerous strategies have been successfully used for the enzyme’s activation and stabilization in IL medium [19], while taking into account which factors affect the enzymatic behavior and how they are affected [27,28]. A considerable number of articles highlighted that the enzyme’s activity and stability is strongly dependent on the physicochemical factors of ILs, such as type of ions, hydrogen-bonding ability and/or hydrophobicity, concentration, and viscosity, among others [3,14,28] Moreover, the same IL can have distinct effects on different enzymes, stressing the importance of the biomolecule as well [22,23].

**Figure 2:**
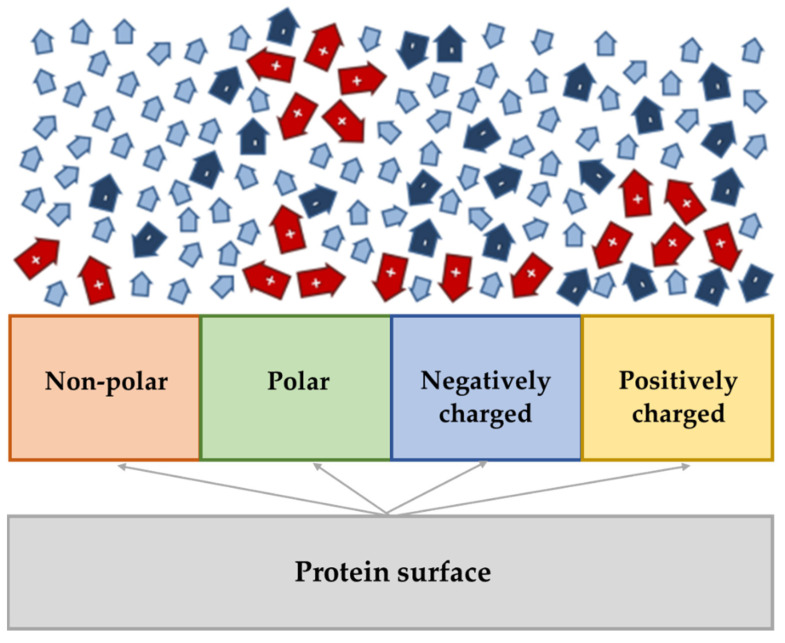
Scheme of the interactions of the dissolved protein in IL-water mixtures. The anions (dark blue arrows) have a preference to the neighborhood of the surface that are positively charged or to the hydration event of water molecules (light blue arrows) in the bulk phase. The cations (red arrows) aggregate in the form of hydrophobic clusters or are pushed to the protein surface. Adapted from reference [19].

The wide structural variety of ILs and proteins/enzymes is responsible for a multiplicity of possible solvent–protein interactions [21], which are summarized in Figure 2 [19]. Several studies have revealed a higher concentration of cations at the protein surface than the one verified for anions, regardless of the charge presented by the protein [26,29,30]. For proteins in aqueous media, this event can be justified by the preferential hydrogen-bonded environment established between IL anions and water molecules in the bulk phase [19]. However, if properly designed, IL cations have an amphiphilic nature, where long alkyl chains provide apolar regions and can interact favorably with the non-polar protein surface moiety [18,26]. Cations (red arrows in Figure 2) compete with the IL anions (dark blue arrows) for solvation positions at the polar protein surface, and in this way, most of the cationic alkyl chains point away from the surface of the protein. Notwithstanding that proteins with negative charge attract more cations in comparison to anions, the latter continue to compete with water molecules (light blue arrows in Figure 2) to establish hydrogen bond interactions with the amino acids present on the surface [19].

Hydrophilic anions generally choose the positively charged protein/enzyme surface, while they attempt to form hydrogen bonds to positively charged amino acids and to establish strong Coulomb interactions. Coulombic interactions established between the IL anions and the protein are stronger in comparison to those verified for the cations [19]. If the interaction of IL ions with water is stronger than with proteins, water molecules are eliminated from the protein surface with increasing IL concentration. This removal can be beneficial or detrimental regarding the solubility of the protein. For instance, hydrophobic ILs may create two phase systems together with water, causing fewer reduction of water from the protein surface and a consequent enhanced solubility of the protein [19].

Numerous studies have demonstrated that the performance of important biomolecules such as proteins and enzymes can be substantially improved by using ILs. Several studies revealed that the functionality of these biomolecules can be improved in the presence of ILs [31,32,33,34,35,36,37,38,39]. Relevant works have been published demonstrating that proper ILs improve the activity and stability of various proteins or enzymes, comprising lysozyme (lyz), laccase, alcohol dehydrogenases, and cytochrome c (cyt-c), among others [17]. 

The competitive benefit in the manufacture of proteins also relies on the innovation and optimization of separation and purification processes [40]. The fact that protein purification bioprocesses are expensive remains a major obstacle of modern biotechnology [41]. Therefore, an efficient and low-cost production of pure and high-quality proteins is crucial [42]. Biocompatibility is a key feature in the design of novel and biocompatible platforms to separate and purify high-value enzymes and proteins [43]. The separation and purification of the target protein in these processes are critical and highly complex tasks because proteins undergo denaturation and lose their biological activity under the usual multistage processes [42,44,45]. In this respect, the employment of proper designed ILs offers a favorable alternative, which has drawn considerable and increasing interest [45]. ILs allow overcoming the drawbacks related to standard processes, such as harsh conditions and toxic volatile organic solvents that could lead to the loss of biological activity of proteins.

Regarding the field of separation and purification of proteins/enzymes, systems based on ILs have attracted the attention as potential platforms in liquid–liquid extractions (using non-water miscible ILs or aqueous biphasic systems (ABS) made by ILs) and in solid–liquid extractions where ILs are attached through covalent interactions (SILs) [46]. IL-based ABS correspond to a particular liquid–liquid extraction type, being created through the addition of salts, carbohydrates, polymers, or amino acids to IL aqueous solutions [47]. Since ABS are mainly composed of water, if properly designed, they offer many relevant advantages: for instance, high extraction efficiency, enhanced selectivity, mild operating conditions, short equilibration time, and a biocompatible environment [47,48]. The effect of the ILs structure and concentration, pH, temperature, and salt are the main parameters studied in the extraction and purification of proteins, such as bovine serum albumin (BSA), cyt-c, lipase, and immunoglobulin G (IgG), among others. As a consequence of the referred advantages, IL-based ABS have been extensively reviewed [47,48,49,50]. BSA corresponds to one of most investigated model proteins, and its extraction efficiency was evaluated using e.g., IL-based ABS formed by tetraalkylphonium- or tetraalkylammonium-based ILs, combined with a potassium citrate/citric acid buffered aqueous solution (pH = 7.0) [44]. The achieved results reveal that except for the ABS formed by more hydrophobic ILs, the systems studied permit the complete extraction of BSA to the phase rich in IL in a single step, while more hydrophobic ILs led to BSA precipitation and/or denaturation at the ABS interface. For the extraction and purification of proteins, more biocompatible ABS have been also studied: for instance, ABS composed of cholinium-based ILs combined with poly(propylene glycol) (PPG), showing a favored migration of BSA to the IL-rich phase and extraction efficiencies of 100% [44,51,52].On the other hand, other less usual IL-based ABS, such as thermoreversible IL-based ABS formed by protic ILs, were studied for the extraction of cyt-c and azocasein [53]. Researchers demonstrated a complete extraction of both proteins, which was accomplished in a single step, through the application of temperature-induced phase switching [53].

Although the largest fraction of works on enzymes and proteins combined with ILs comprises their use in liquid–liquid extraction processes, relevant evidence has been published on SILs’ potential in similar fields, i.e., as novel enzymatic supports and in proteins/enzymes purification, which are here overviewed. 

## 2. Supported Ionic Liquids (SILs)

It was in the 1970s–1980s that the supported liquid phase field started [54], being achieved by the physical deposition of a liquid onto a material. However, since then, many studies disclosed the evaporation of the loaded liquid, which was a particular problem, especially when applying water as the liquid phase, making the concept appropriate merely for slurry-phase reactions with hydrophobic reaction mixtures [54]. Thus, new approaches to obtain liquid-comprising solid materials that do not have the ability to evaporate have emerged, particularly by modification of the surface of a porous solid through dispersion of a thin film of IL, which was described as supported ionic liquids (SILs). SILs are a particular group of materials obtained by the immobilization of ILs in a suitable solid support [55]. SILs present several advantages such as adjustable solvent properties with an efficient immobilization on a confined environment [55]. Since IL properties are transferred to solid surfaces, the SIL concept allows creating and customizing the solid material (‘‘designer surfaces’’), resulting in a surface that is well-defined and presents new specific properties and regulated chemical reactivity, thus constituting an attractive methodology to traditional material science [54]. 

Depending on the nature of the interactions settled between the IL and the support material, two principal categories can be distinguished: (i) the physical confinement of the IL in the materials (physisorption); and (ii) covalent grafting (chemisorption), in which covalent bonding between the support and the IL exists [55,56,57]. In the physisorption method, the immobilization of ILs occurs through deposition with non-covalent binding involved. Usually, SILs obtained by this method are present as a monolayer, although multilayers or brushes can also be obtained [58]. In this type of SIL, the properties of neat ILs are almost preserved. These materials have been mainly investigated in the capture and separation of gases [59,60,61]. On the other hand, immobilizing ILs on materials through covalent attachment is an attractive approach to avoid ILs leaching that may occur in the physical deposition method [62]. However, in these materials, the specific properties noted in the IL bulk phase may no longer be present in the prepared support, since there is not a liquid phase present [62]. Despite this condition, the IL chemical structure diversity and their designer solvents ability are translated into SILs, contributing to enhancing the selectivity as well as the adsorption ability of the material in target applications [62,63].

Due to the recognized advantages obtained with SILs, numerous applications in different areas, varying from chemical to biological sciences, have been proposed [55]. In their use in solid-phase extractions from liquid phases or in biocatalytic applications, SILs with ILs covalently attached are preferred, since they avoid leaching. Solid–liquid systems exhibit numerous advantages in comparison to classical gas–liquid or liquid–liquid systems, such as an elevated high surface area provided by the support material, a thin film composed of liquid that avoids mass transport issues, and their application in fixed-bed or fluidized-bed reactor technologies. However, the preparation of this type of SIL exhibits some limitations and drawbacks: (1) a more challenging preparation since at least one reaction step is necessary, additionally including the pre-treatment requirement of inert materials; and (2) usual low density of IL ions onto the support, while being an IL monolayer [64].

## 3. SILs Applications in Proteins and Enzymes-Related Fields

As previously mentioned, ILs can be tailored for target applications by correctly designing their chemical structure. Therefore, the use of biomolecules (proteins/enzymes) together with ILs has been increasingly studied over the years [31]. However, the use of pure ILs in certain processes brings some disadvantages. As an example, in catalysis, their high viscosity makes it difficult to achieve maximum product yield [63]. On the other hand, the disposal of the recovered contaminated IL and associated economic costs are hurdles to overcome [63]. In this way, researchers have been dedicated to the immobilization of ILs onto supports to overcome these drawbacks. 

Up to date, SILs have been broadly employed in almost all fields involving ILs and have led to expansions on the ILs arena [13,62]. The following sections cover the use and application of SILs as alternative supports for enzymes in biocatalysis and in the purification of proteins and enzymes.

### 3.1. SILs in Biocatalysis

**Figure 3:**
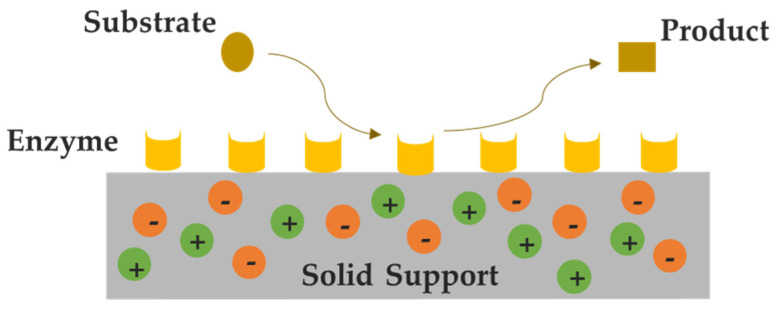
Schematic representation of a biocatalytic system based on SIL. IL is confined in the solid support (gray), whereas the enzyme (yellow) is immobilized over the modified support.

The idea of liquid (bio)catalysis is not limited to homogeneous liquid media, being supported liquid phases an alternative. The novel concept of SILs for catalytic reactions was firstly depicted in 2002 by Mehnert et al. [65] for hydroformylation catalytic reactions. As a result of the rise in the amount of accessible active sites of the catalyst and reduction of mass transfer restrictions, SILs permit an effective usage of the catalyst and a reduced amount of IL needed [55]. This possibility decreases the economic cost and possible environmental pressure in an enzymatic process of large-scale. In addition, the growing concerns about their toxicological and ecotoxicological properties can be overcome, at least partially and if properly designed, by the use of SILs [15]. In this field, organic and inorganic supports modified with ILs, such as ceramics, macroporous membranes and monoliths, magnetic nanoparticles celite, resins, and polymers have been used for the immobilization of enzymes [66,67,68]. The attachment of the enzyme can be carried out through the adsorption or chemical bonding of the free enzyme in an aqueous solution on the SILs surface. The type of IL, the amount of IL, and its distribution on the support surface area is of vital importance to preserve and improve the biocatalytic performance [55]. Figure 3 shows a schematic representation of a biocatalytic system based on SIL.

The studies described in the literature proving SILs’ applicability for biocatalysis purposes are compiled in Table 1. SILs were employed to immobilize *Candida Antarctica* Lipase B (CALB) in a hybrid monolith [69]. Therefore, a Teflon^®^ cartridge along with porous cellulose-2.5-acetate beads were used to prepare the monolith. Then, the IL 1-octyl-3-methylimidazolium tetrafluoroborate, comprising small amounts of CALB, was impregnated into the referred beads, leading to the formation of an enzyme-supported ionic liquid phase (e-SILP). Then, a continuous gas-phase transesterification reaction of 2-propanol and vinyl propionate was performed using the immobilized enzyme, with conversions > 30% after 24 s [69]. In a similar work, bioreactors consisting of a monolith-supported IL stage, with covalently bonded IL-like moieties, were prepared for enzyme catalysis in supercritical CO_2_ [70]. The monolith-supported IL phase with adsorbed CALB was assessed for the continuous flow synthesis of citronellyl propionate in supercritical CO_2_ conditions. The bioreactor performance remained unchanged during seven reaction cycles of 5 h each. The best results were acquired when the most hydrophobic monolith, M-SILP-8-CALB, was assayed at 80 °C and 10 MPa, achieving a total turnover number (TON) of 35.8 × 10^4^ mol product/mol CALB [70,71]. Different CALB–SILLPs with distinct covalently attached IL-like moieties were developed by the same research group, to be used as biocatalysts for a stereospecific reaction model, for instance the kinetic resolution (KR) of rac-1-phenylethanol in both batch and continuous process. These biocatalysts exhibit good performance in a traditional organic solvent (hexanes) and in supercritical CO_2_. Additionally, the continuous one-pot dynamic kinetic resolution (DKR) of this substrate in supercritical CO_2_ conditions through in situ racemization of the undesired enantiomer was also described with good chemical yield (>85%) and excellent enantiopurity, above 99% [71]. In a different work, a SILP was prepared by adsorption, i.e., via non-covalent interactions, of 1-butyl-3-methylimidazolium hexafluorophosphate ([bmim]PF_6_]) and 1-butyl-3-methylimidazolium tetrafluoroborate ([bmim]BF_4_]) ILs on a single-walled carbon nanotubes (SWNT) surface [72]. Then, this SIL was applied in the immobilization of proteins containing a heme group and enzymes, namely cyt-c, myoglobin, as well as horseradish peroxidase for bioelectrocatalytic activities. The immobilized enzymes on the IL-SWNT composites retained their bioelectrocatalytic activity to the decrease in oxygen and hydrogen peroxide, demonstrating a novel approach for proteins/enzymes electrochemistry, electrocatalysis, and bioelectrochemical synthesis. Accordingly, these biocomposites have promising applications in the development of biofuel cells, biosensors, and other bioelectrochemical devices [72]. 1-Butyl-3-methylpyridinium tetrafluoroborate ([bmpyr]BF_4_), 1-butyl-1-methylpyrrolidinium tetrafluoroborate ([bmpyrr]BF_4_), and [bmim]BF_4_] ILs were applied to modify the SWNTs surface and immobilize glucose oxidase (GOx), aiming to evaluate the oxidation of glucose via electrochemistry and quantum chemistry calculation [73]. ILs and GOx were assembled on the SWNTs surface through electrostatic interactions. According to spectroscopic studies, ILs did not impact GO conformation. The characteristic rate constant values obtained at each IL-comprising enzyme electrode decreased according to the following sequence of SWNTs: [bmpyr] > [bmpyrr] > [bmim]. Relevant calculations along with experiments were used to address the interactions established between ILs and SWNTs, showing that the presence of the IL on the surface of the referred material could significantly affect the electrical transfer properties exhibited by the nanotubes and lead to the reduction of the electrocatalytic activity of the electrode. These results suggest that the nature of ILs is the main factor impacting the electrocatalytic activity of the electrodes toward glucose oxidation [73].

Another type of carbon nanotubes, namely multiwalled carbon nanotubes (MWNTs), suffered a covalently modification with imidazolium-based ILs with distinct alkyl groups (ethyl, butyl, octyl, and lauryl) [74]. These SILs were used to immobilize CALB to carry out the hydrolysis of triacetin [74]. In contrast to the immobilized CALB on pristine MWNTs, the CALB immobilized on novel SILs presented superior activity, thermal stability, as well as reusability. Particularly, MWNTs-IL (8C)-CALB activity increased 15.23-fold when compared to MWNTs-CALB. Following incubation at 70 °C for 20 min, the residual enzyme activity of MWNTs-IL (8C)-CALB was 46% of the initial one, while MWNTs-CALB lost all activity. Moreover, MWNTs-IL (8C)-CALB maintained 64.5% of its initial activity after four cycles, while MWNTs-CALB retained only 2.12% [74]. 

Phosphonium-based ILs were used as well to prepare and non-chemically modify silica for further use as supports for lipase immobilization. Several phosphonium-based ILs were studied, namely tetrabutylphosphonium chloride ([P_4444_]Cl), trihexyltetradecylphosphonium chloride ([P_666(14)_]Cl), tributyltetradecylphosphonium chloride ([P_444(14)_]Cl), trihexyltetradecylphosphonium decanoate ([P_666(14)_][Deca]), trihexyltetradecylphosphonium bromide ([P_666(14)_]Br), trihexyltetradecylphosphonium bis(2,4,4-trimethylpentyl)phosphinate ([P_666(14)_][Phosp]), and trihexyltetradecylphosphonium bis(trifluoromethylsulfonyl)amide ([P_666(14_)][NTf_2_]) [75]. The results gathered by the authors prove that the enzymatic performance of lipase was influenced by the IL used to prepare silica, in which a positive effect is noted when ILs containing cations with longer alkyl side chains and more hydrophobic anions were used. [P_666(14_)][NTf_2_] was the best IL, allowing a relative activity of 209.8% and an immobilization yield of 77.3%, with a recycling capacity of eight times, while keeping more than 50% of the initial activity of the enzyme [75]. 

A carboxyl-functionalized imidazolium tetrafluoroborate IL was used for the covalent modification of a mesoporous silica SBA-15 to immobilize porcine pancreatic lipase through physical adsorption and covalent attachment for the hydrolysis reaction of triacetin [76]. It was found that SILs are effective to enhance the properties of the immobilized enzyme, namely by providing more resistance to temperature and pH changes. Moreover, the covalent attachment to the SIL leads to a retention of 81.25% and 52.50% of the initial enzyme activity after 20 days of incubation and four cycles of reuse, respectively. On the other hand, the physical adsorption of IL led only to 58.80% and 27.78% of the initial enzyme activity [76]. 

Among the studied supports, magnetic nanoparticles (MNPs) are also an ideal support for enzymatic reactions because of their high specific surface area, as well as their magnetic properties, and thus easier recovery by applying magnetic fields. The combination of ILs and magnetic carboxymethyl cellulose (IL-MCMC) is an ideal approach for the development of modified nanoparticles to be applied as supports for the immobilization of the enzyme [77]. Suo et al. [77] used IL-MCMC as a support for lipase immobilization; the specific activity of immobilized lipase was 1.43- and 2.81-fold higher than that verified for the free lipase and for the lipase immobilized on MCMC, respectively. Results regarding the contact angle revealed that the ILs insertion enhanced the carrier hydrophobicity, which induced the lid-opening of lipase, resulting in a more accessible enzyme active site. The same IL-MCMC was evaluated in penicillin G acylase (PGA) immobilization to validate the broader method applicability. The results indicated that the immobilized PGA presented higher stability in comparison to many other reported materials [77]. ILs were also used to modify magnetic chitosan (MCS) composites to be applied as supports for Porcine pancreatic lipase (PPL) adsorption, with graphene oxide (GO) used as a shell coating [78]. In this work, an imidazolium-based IL with a side alkyl chain formed by eight −CH_2_ and a terminal hydroxyl group was used. The prepared SIL was able to maintain high PLL activity (2468 U/g), which was 6.72-fold higher compared to free lipase. Additionally, following 10 cycles of reuse, the residual activity of the immobilized enzyme was 92.1%. Circular dichroism was employed to prove that the secondary structure of the enzyme was kept stable [78]. 

The 1-decyl-2-methyimidazolium cation was covalently bound to a polystyrene divinylbenzene porous matrix to immobilize CALB for biodiesel synthesis (methyl oleate) through the methanolysis of triolein in the presence of tert-butanol and supercritical (sc)CO_2_ [79]. The immobilized enzyme on the SIL resulted in the best biodiesel yields, >95 %, and operational stability (biodiesel yield of 85 % after 45 cycles of 8–4 h) in the presence of (sc)CO_2_. Furthermore, the presence of tert-butanol was crucial to prevent poisoning of the biocatalyst through the blockage of its active sites by the polar by-product (glycerol) produced during biodiesel synthesis [79]. The acylation of 1-phenylethanol with vinyl acetate in hexane and in toluene was carried out using lipase from *Burkholderia cepacia* immobilized on a Kynol™ ACC 507-15 modified with 1-ethyl-3-methylimidazolium bis(trifluoromethanesulfonyl)imide ([emim][NTf_2_]), 1-ethyl-3-methylimidazolium tetrafluoroborate ([emim][BF_4_]), methyltrioctylammonium trifluoroacetate ([mtoa][TFA]), 1-butyl-4-methylpyridinium tetrafluoroborate ([mbpyr][BF_4_]), and 1-butyl-3-methylimidazolium trifluoromethanesulfonate ([bmim][TfO]) [80]. According to the obtained results, the modification with [emim][NTf_2_] leads to a more stable enzyme against inactivation, besides preserving its enantioselectivity in reuse.

Giacalone et al. [58] showed that with CALB, the biological activity can be increased by supporting the enzyme onto an SIL. In this study, the immobilization of CALB was carried out by using a poly(styrene-co-divinylbenzene) support, a macroporous resin, with low and high degrees of functionalization. The resins were covalently modified through reaction with 1-dodecyl-2-methylimidazole. Then, CALB was immobilized through adsorption from an aqueous solution of the enzyme onto the SIL bearing chloride (Cl^−^) or bis(trifluoromethanesulfonyl)imide ([NTf_2_]^−^) as the counterion [58]. A substantial improvement of the enzyme activity was noted for the enzyme supported on the material. The authors obtained a 28-fold increase for CALB supported on the SIL comprising the hydrophobic [NTf_2_]^−^ counter anion with a low level of functionalization, under microwave irradiation [58]. However, a greater concentration of IL-like fragments has an opposite effect [58]. 

Overall, in the area of SILs employed in biocatalysis applications, the amount of IL, the distribution of the latter on the support material, and the nature of the cation or counterion are considered essential for the maintenance or improvement of the intrinsic catalytic properties of the enzyme. Despite the promising results described, there is still a long path to cross in this field, particularly considering the matrix of materials, enzymes, and ILs that can be combined.

### 3.2. SILs as Separation and Purification Platforms for Proteins and Enzymes

**Figure 4:**
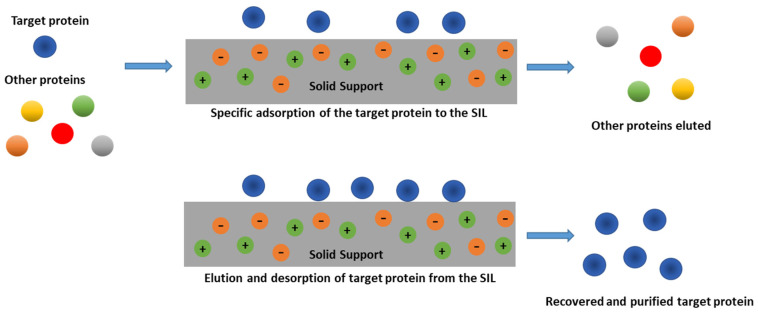
Schematic representation of an SIL-based system for proteins/enzymes purification. The IL is confined in the solid support (orange/gray), either physically deposited or chemically bound at the material surface. Proteins adsorb at the surface material according to specific interactions (hydrophobic, hydrogen bonding, or electrostatic interactions, or by an ion exchange phenomenon) established with the IL ions.

Immobilized ILs have also been used in the area of separation science. Continuing to take advantage of the ILs exceptional features, SIL materials have been projected as new chromatographic matrices in separation processes, which could be utilized as new chromatographic columns or in solid-phase extraction (SPE) strategies [81]. SPE is a purification process centered on solid materials used as affinity stationary phases to provoke the adsorption of target molecules from the liquid extracts, and it corresponds to the most broadly used sample-preparation methodology for liquid samples. Moreover, the main advantages exhibited by SPE are efficiency and low reagent consumption. Thus, the utilization of ILs as SPE adsorbents for protein separation purposes, which can include preparative liquid chromatography, is a promising application. Furthermore, protein separation is one of the fundamental primary steps of proteomics experiments, since biological samples are complex matrices and the coexisting components seriously affect the detection of individual proteins [82]. Accordingly, SILs have been effectively applied in the separation of proteins and enzymes with reasonably high adsorption capacity and specific recognition [46]. Moreover, imidazolium-based ILs have been applied in capillary electrophoresis to enhance the electroosmotic flow and proteins separation [83,84,85]. Figure 4 depicts a schematic representation of an SIL-based system for proteins/enzymes purification.

SILs have been studied in SPE in order to take advantage of the chemical versatility and tunability of ILs. Most studies with SILs in this field used silica or polymers as solid phases. However, this field is less developed than the use of SILs in biocatalysis, with their application in separation processes of proteins being still limited for standard proteins such as lyz, BSA, bovine hemoglobin (BHb), cyt-c, ovotransferrin (OVT), and hemoglobin (Hb). The findings reported in the literature proving the SILs capability in the separation/recovery of some proteins are given in Table 2.

Shu et al. [86] prepared [Nmim]Cl/polyvinyl chloride (PVC) materials by the immobilization of imidazolium cations, namely [Nmim]^+^ moieties onto the PVC chain. Through this new method, [Nmim]Cl/PVC IL at a molar ratio of 4:1 gives rise to a bulk immobilization ratio of 15.1%. These SILs were able to perform the adsorption of lyz, cyt-c, as well as Hb with high efficiencies, viz. 97%, 98%, and 94%, respectively [86]. It was also pointed out by the authors that the prepared hybrid material exhibited potential properties crucial for its separation performance: for instance, an outstanding selectivity to adsorb basic proteins through an effective suppression of the adsorption of non-specific proteins by the pure PVC material, as well as its enhanced biocompatibility that avoids protein denaturation phenomenon during adsorption. In a different work, the selective isolation of Hb was assessed using imidazolium-modified polystyrene (PS) materials [87]. Imidazolium cations ([mim]^+^) were attached onto the chloromethyl polystyrene (PS-CH_2_-Cl) surface, forming PS-CH_2_-[mim]Cl, which is a crosslinked rigid polymer that can behave as a support. The adsorption efficiencies of this material to Hb achieved values up to 91%, with almost no protein denaturation detected [87]. It was also stressed by the authors that the immobilization of the imidazolium groups on the surface of the material showed potential properties crucial for its separation performance, such as the ability to suppress the non-specific protein adsorption, promoting the selective adsorption of Hb. Another two polymer materials were synthesized and applied for the separation of BSA, BHb, lyz, and cyt-c, in which 1-allyl-3-butylimidazolium chloride ([aC_4_im]Cl) and 1-vinyl-3-octylimidazolium bromide ([VC_8_im]Br) were employed as functional monomers, and acrylamide was employed as a co-functional monomer [88]. While the [aC_4_im]Cl-based polymer material has a high binding capacity for BHb (828.5 mg g^−1^), the [VC_8_im]Br-based polymer material has it for BSA (804.7 mg g^−1^) [88]. In a similar work using a macroporous polymer material modified with 1-vinyl-3-butylimidazolium chloride ([ViBuIm]Cl), the selective adsorption of five proteins, namely BSA, BHb, equine myoglobin, cyt-c, and lys, was evaluated **[91]**. The adsorption tests included the adsorption capacity using individual proteins, binary mixtures composed by lyz and BSA, and lyz and cyt-c, as well as a ternary mixture made of lyz, BSA, and cyt-c. The prepared SIL displayed a strong binding capacity for proteins, in particular for Lys, registering a maximum capacity of 755.1 mg g^−1^. Regarding the mixture of proteins, BSA was acknowledged as a competitive protein in the referred binary and ternary protein solutions, as the amount of adsorbed BSA was greater than the amount of adsorbed lyz. In addition, the amount of adsorbed BSA from the mixtures was greater in comparison to that of only BSA without other proteins, while the amount of adsorbed lyz of the mixtures was lower compared to that of only lyz without other proteins [91]. On the other hand, when the mixtures exhibit cyt-c, the amount of adsorbed lyz and cyt-c decreased when compared to only one protein in the solution due to the competitive adsorption that exists between the two proteins (with similar molecular weight and pI). Comparing the ternary solution composed of lyz, BSA, and cyt-c and the solution containing only cyt-c, the differences in the adsorption capacities of cyt-c were not obvious, suggesting that the interaction between proteins does not present a significant effect on cyt-c adsorption. The authors concluded that electrostatic interactions, hydrophobic forces, as well as hydrogen bonding were the main interactions established between proteins and the SIL [91]. 

A diversity of polymeric IL materials with the capacity of adsorbing different proteins were prepared with 2-acrylamido-2-methylpropane sulfonic acid imidazole derivative [C_1_C_3_S][Rim]-MBA (R: –H, –C_6_H_5_, or –(CH_2_)_3_CH_3_) acting as a functional monomer [94]. The results showed, at pH 4.0, a high adsorption capacity for BSA (842.2 mg g^−1^) and ovotransferrin (OVT, 861.5 mg g^−1^), whilst for cyt-c (882.9 mg g^−1^) and BHb (983.4 mg g^−1^), the higher adsorption occurs at pH 7.0 and at pH 8.0 for casein (605.2 mg g^−1^). Furthermore, the material was prone to separate BHb from a real matrix of bovine blood [94], demonstrating the relevant potential of SILs to deal with complex matrices. 

IL-functionalized nanoparticles (SiO_2_@IL) were prepared through a one-step grafting reaction, using a silane-coupling IL, 1-(3-trimethoxysilylpropyl)-3-methylimidazolium chloride ([tmim]Cl) [93]. The resultant SiO_2_@IL was able to adsorb, at pH 7, a maximum BSA amount of 23.1 mg g^−1^, in which, through experimental tests and theoretical studies of density functional theory (DFT), electrostatic interactions were considered the main driving force [93]. The acidic IL 1-methylimidazolium hydrogen sulphate ([mim][HSO_4_]) supported IL was used to modify silica gel (SiO_2_@[mim][HSO_4_]) and applied in BSA adsorption and separation [92]. The BSA adsorption onto [mim][HSO_4_] followed the Langmuir adsorption equilibrium model. Thermodynamic parameters such as the change of enthalpy, entropy, and Gibbs free energy suggested that the adsorption of BSA was an exothermic and spontaneous process controlled by both chemical and physical interactions [92]. All proteins analyzed achieved their maximum adsorbed amount on SiO_2_@[mim][HSO_4_] when the pH value was around their isoelectric point. Moreover, the capability of SiO_2_@[mim][HSO_4_] to be reused was proved following the protein desorption by using an NaCl aqueous solution. At optimized conditions, it was possible to obtain a selective BSA and BHb adsorption from cow blood [92].

IL-modified magnetic nanoparticles (ILs-MNPs) were recently suggested to recover BSA through the attachment of an hydroxy functional IL to the silica-coated magnetic Fe_3_O_4_ surface, leading to extraction efficiencies for BSA of 86.9% [89]. In addition, the BSA recovery from the material was studied, and it was found that with NaCl at concentrations higher than 1.1 mol L^−1^, the BSA desorption ratio achieves a value of 95.3% [89]. Furthermore, nearly 95% of the prepared ILs-MNPs were recovered, without significant losses on their adsorption efficiency after four cycles [89]. Lately, Wen et al. [90] examined the performance of an amino functional dicationic IL (AFDCIL) coated on the surface of magnetic graphene oxide (Fe@GO) to act as a magnetic adsorbent (Fe@GO@AFDCIL) for BHb protein. The Fe@GO@AFDCIL composite has outstanding adsorption and exhibited higher extraction capacity for BHb compared to traditional Fe@GO@IL composites. Additionally, Fe@GO@AFDCIL revealed good stability, showing the ability of being reused at least 15 times without a considerable decrease in its extraction capacities (<12.5%). These results indicated that the referred magnetic adsorbent could be successfully applied in the extraction of bovine Hb from real samples [90]. The high adsorption capacity, selectivity of the adsorption, and reusability make these systems promising materials for a broad variety of applications. 

Despite the described promising results, more studies must be carried out to extend the range of proteins and complexity of the matrices used to fully appraise their potential. However, the described outcomes, although still scarce in the field of proteins separation, indicate that different materials can be designed to adsorb and separate specific proteins and therefore can be seen as potential approaches to separate target proteins from complex real matrices. So far, studies addressing the purification of target proteins and enzymes from real and complex matrices only addressed bovine serum samples. There are also simulation studies on SILs; most of them focused on the absorption and separation of gases [95,96]. These studies could open the door for the scientific community to start carrying out simulation studies of IL-based materials applied to the protein/enzyme separation and purification fields and thus better understand the molecular-level phenomenon of solid-phase extraction and favorable interactions to improve the material selectivity. The use of IL-based materials for protein separation is yet at an early phase, but this is a novel area where significant progress is estimated in the coming years, confirming the high potential of these materials in proteins downstream processing. 

## 4. Conclusions

The development of novel IL-based materials has shown to be a field of high interest over the last few years. In the field of proteins and enzymes, SILs have been used as physisorbed or chemisorbed ILs in materials, while they have also been applied as enzyme supports in biocatalysis and in the separation of proteins and enzymes. IL-modified materials such as polymeric (hybrid) monoliths, single-walled carbon nanotubes, multiwalled carbon nanotubes, silica gel and mesoporous silica SBA-15, magnetic carboxymethyl cellulose, magnetic chitosan composites, polystyrene divinylbenzene porous matrix, Kynol™ ACC 507-15, poly(styrene-co-divinylbenzene), polyvinyl chloride, polystyrene, and magnetic graphene oxide, among others, have been used. On the other hand, the proteins and enzymes studied in these fields correspond to lysozyme, hemoglobulin, bovine serum albumin, bovine hemoglobulin, equine myoglobulin, Candida antarctica lipase B, myoglobin, horseradish peroxidase, glucose oxidase, *Burkholderia cepacia* lipase, porcine pancreatic lipase, lipase, and penicillin G acylase. 

Although the field of biocatalysis and SILs is a well-known and evolving field, the field of proteins separation/purification is still an underexplored area given the plethora of available material types, ILs, and proteins/enzymes. Furthermore, there is a critical need to start working with real and complex biological matrices in the field of proteins separation, as well as to apply spectroscopic and computational tools to better understand the main factors ruling selectivity and adsorption efficiency. On the other hand, these separation techniques based on SILs should be applied as well to separate high-value proteins, as is the case of biopharmaceuticals. Accordingly, it is expected that in the next few years, more investigations will be accomplished, and promising new results in this specific area will be disclosed.

## Figures and Tables

**Table 1:** Summary of the reported studies showing the potential of SILs in the biocatalysis field.

Support(s)	Immobilization Type	Enzyme(s)	Reaction/Application	Reference
Polymeric hybrid monolith	Physisorption	CALB	Vinyl propionate and 2-propanol continuous gas-phase transesterification	[69]
Polymeric monolith	Physisorption	CALB	Continuous flow synthesis of citronellyl propionate in supercritical CO_2_ conditions	[70]
Polymers	Physisorption	CALB	Biocatalysts for a stereospecific reaction model	[71]
Single-walled carbon nanotubes (SWNT)	Physisorption	Myoglobin, cyt-c and horseradish peroxidase	Bioelectrocatalysis	[72]
Single-walled carbon nanotubes (SWNTs)	Physisorption	Glucose oxidase (GOx)	Oxidation of glucose	[73]
Multiwalled carbon nanotubes (MWNTs)	Physisorption	CALB	Hydrolysis of triacetin	[74]
Silica	Physisorption	*Burkholderia cepacia* lipase	Supports for lipase immobilization	[75]
Mesoporous silica SBA-15	Physisorption and chemisorption	Porcine pancreatic lipase	Hydrolysis of triacetin	[76]
Magnetic carboxymethyl cellulose (IL-MCMC)	Chemisorption	Lipase and penicillin G acylase (PGA)	Supports for enzyme immobilization	[77]
Magnetic chitosan (MCS) composites	Physisorption	Porcine pancreatic lipase (PPL)	Supports for PLL adsorption	[78]
Polystyrene divinylbenzene porous matrix	Physisorption	CALB	Synthesis of biodiesel	[79]
Kynol™ ACC 507-15	Physisorption	*B. cepacia* lipase	1-Phenylethanol acylation with vinyl acetate	[80]
Poly(styrene-co-divinylbenzene)	Physisorption	CALB	Catalysis	[58]

**Table 2:** Summary of the reported studies showing the potential of SILs to separate/recover proteins.

Material(s)	Protein(s)	Observation	Reference
[Nmim]Cl/polyvinyl chloride (PVC)	lyz, cyt-c and Hb	Materials were able to adsorb proteins with high efficiency	[86]
Imidazolium-modified polystyrene	Hb	High adsorption efficiencies for Hb, with almost no protein denaturation using these materials	[87]
1-Allyl-3-butylimidazolium chloride ([aC_4_im]Cl) and 1-vinyl-3-octyl-imidazolium bromide ([VC_8_im]Br) based polymers	BSA, bovine hemoglobin (BHb), lyz, and cyt-c	Different materials can be designed to adsorb and separate specific proteins	[88]
IL-modified magnetic nanoparticles (ILs-MNPs)	BSA	High adsorption capacity, selective adsorption, and stability of BSA	[89]
Amino functional dicationic IL (AFDCIL)/magnetic graphene oxide (Fe@GO)	BHb	Excellent adsorption and high extraction capacity for BHb	[90]
1-vinyl-3-butylimidazolium chloride ([ViBuIm]Cl) macroporous polymer	BSA, BHb, equine myoglobin, cyt-c and lys	The macroporous IL polymer material presents an excellent adsorption capacity for proteins, especially for lyz	[91]
Silica gel functionalized with IL (SiO_2_@IL) IL: 1-methylimidazolium hydrogen sulphate ([mim][HSO_4_^−^])	BSA, BHb	At the optimized conditions, a selective adsorption of BSA and BHb was successfully obtained from cow blood	[92]
Silane-coupling IL (SiO_2_@IL) IL: 1-(3-trimethoxysilylpropyl)-3-methylimidazolium chloride ([tmim]Cl	BSA	The SiO_2_@IL is able to adsorb BSA, being the main driving force of electrostatic interactions	[93]

## Data Availability

Not applicable.
